# Comprehensive analysis of spread through air spaces in lung adenocarcinoma and squamous cell carcinoma using the 8th edition AJCC/UICC staging system

**DOI:** 10.1186/s12885-020-07200-w

**Published:** 2020-07-29

**Authors:** Meng Jia, Shili Yu, Jiaqi Yu, Yuemin Li, Hongwen Gao, Ping-Li Sun

**Affiliations:** Department of pathology, The Second Hospital of Jilin University, 218 Ziqiang Road, Changchun, 130041 Jilin China

**Keywords:** Non-small cell lung cancer, Adenocarcinoma, Squamous cell carcinoma, Spread through air spaces (STAS), 8th edition AJCC/UICC staging system

## Abstract

**Background:**

This study aimed to comprehensively investigate the effect of spread through air spaces (STAS) on clinicopathologic features, molecular characteristics, immunohistochemical expression, and prognosis in lung adenocarcinomas (ADC) and squamous cell carcinomas (SQCC) based on the 8th edition AJCC/UICC staging system.

**Methods:**

In total, 303 ADC and 121 SQCC cases were assessed retrospectively. Immunohistochemical staining was performed for E-cadherin, vimentin, Ki67, survivin, Bcl-2, and Bim. Correlations between STAS and other parameters were analyzed statistically.

**Results:**

STAS was observed in 183 (60.4%) ADC and 39 (32.2%) SQCC cases. In ADC, the presence of STAS was associated with wild-type EGFR, ALK and ROS1 rearrangements, low E-cadherin expression, and high vimentin and Ki67 expression. In SQCC, STAS was associated with low E-cadherin expression and high vimentin and survivin expression. Based on univariate analysis, STAS was associated with significantly shorter disease-free survival (DFS) and overall survival (OS) in ADC. In SQCC, STAS tended to be associated with shorter OS. By multivariate analysis, STAS was an independent poor prognostic factor in ADC for DFS but not OS. Stratified analysis showed that STAS was correlated with shorter DFS for stage I, II, IA, IB, and IIA ADC based on univariate analysis and was an independent risk factor for DFS in stage I ADC cases based on multivariate analysis.

**Conclusions:**

Our findings revealed that STAS is an independent negative prognostic factor for stage I ADC using the new 8th edition AJCC/UICC staging system. Stage I patients with STAS should be followed up more closely and might need different treatment strategies.

## Background

Spread through air spaces (STAS) is a phenomenon of lung cancer spread, which is defined as tumor cells within air spaces in the lung parenchyma beyond the edge of the main tumor. STAS was first named by Kadota and colleagues in 2015 [1] and has received widespread attention since its identification. The significance of STAS is predominantly due to its predictive value on prognosis. The presence of STAS was found to be correlated with aggressive clinicopathologic features and poor prognosis in several histological types of lung cancers. Moreover, according to 2015 World Health Organization (WHO) classification [2], this morphological manifestation was listed as an exclusion criterion for the diagnosis of adenocarcinoma in situ and minimally invasive adenocarcinoma (MIA). Although the clinicopathologic features and prognostic significance of STAS have been investigated, the published studies were mainly conducted according to the 7th edition of American Joint Committee on Cancer (AJCC)/Union for International Cancer Control (UICC) staging system; few studies have analyzed the association between STAS and pathological stage (p-stage) using the new 8th edition AJCC/UICC staging system. Compared with the 7th edition of AJCC/UICC staging system, the change in the new TNM staging criteria mainly concerns the description of T. T stage is subdivided at a 1-cm cut-off when the tumor size is less than or equal to 5 cm [3], and this improved T staging results in a better correlation with prognosis. However, although STAS has been reported to be significant with respect to the prediction of survival for early-stage tumors, few studies have analyzed the significance of STAS based on a single subdivided stage exclusively.

In addition to the aforementioned challenges, the association between STAS and molecular characteristics of lung adenocarcinoma (ADC) has not been clearly explicated, and this issue has been barely studied in Chinese patients. Meanwhile, little progress has been achieved in elucidating the association between STAS and the immunohistochemical expression of epithelial–mesenchymal transition (EMT), proliferation, and apoptosis-related markers. The purpose of this study was to comprehensively investigate the effect of STAS on clinicopathologic features, molecular characteristics, immunohistochemical expression, and prognosis in lung ADC and squamous cell carcinomas (SQCCs) based on the 8th edition AJCC/UICC staging system.

## Methods

This study was approved by the ethics committee of The Second Hospital of Jilin University (Changchun, China). Written informed consent was also obtained from all patients.

### Patients and sample collection

We retrospectively collected the data and tissue specimens of patients who underwent surgical resection (limited resection or lobectomy) for primary lung ADCs and SQCCs between 2010 and 2014. In our institution, limited resection (including wedge resection and segmentectomy) was performed based on a comprehensive consideration of the following issues: (1) tumors smaller than 3 cm with radiologically ground glass node (consolidation/tumor ratio < 0.5); (2) tumor location within the outer third of the lung parenchyma; (3) general status and respiratory function of the patients. Cases with neoadjuvant therapy, positive surgical margins, a diagnosis of multiple primary lung cancers, a diagnosis of in situ or MIA, and no available tumor slides for review were excluded from this study. In total, 303 cases of ADCs and 121 cases of SQCCs were assessed. Clinical parameters including patient age, sex, smoking history, tumor size, p-stage, and follow-up were collected from the original medical records. The tumor p-stage was restaged using the 8th edition AJCC/UICC staging system. The period of follow-up ranged from 1 to 65 months.

### Histological review

All tissue specimens were reviewed retrospectively. Pathological parameters including pleural invasion, blood and lymphatic vessel invasion, perineural invasion, and necrosis were recorded. For ADCs, comprehensive histologic subtyping was also performed. ADCs were classified as lepidic, acinar, papillary, micropapillary, or solid subtypes according to the 2015 WHO classification [2].

Tumor STAS was defined according to the descriptions summarized by Kadota et al. [1]. In each case, at least four slides were observed to detect STAS. The presence of STAS was recorded as “present” or “absent,” regardless of the subtypes of STAS cells. Artificial fragments and other mimics including a micropapillary pattern of invasion and intra-alveolar macrophages were strictly evaluated and excluded.

### Immunohistochemistry

Immunohistochemical staining was performed automatically using PT Link Pre-Treatment system (DAKO, CA, USA) and Autostainer Link 48 system (DAKO, CA, USA). Endogenous peroxidases were quenched with 3% H_2_O_2_ for 10 min. The sections were incubated with primary antibodies (Additional file 1) for 30 min. The samples was then incubated with the secondary biotinylated antibody for 20 min. The slides were stained using 3, 3′-diaminobenzidine and counterstained with hematoxylin.

### Scoring of immunostained tissue sections

The expression of markers was quantified based on the extent of staining (by percentage of positive tumor cells: 0–100%; for E-cadherin, only tumor cells with complete membranous staining were counted) and the intensity of staining (graded on a scale of 0–3 as follows: 0, no staining; 1, weak staining; 2, moderate staining; and 3, strong staining). A semi-quantitative score was obtained by multiplying the grades of intensity by the percentage of positively stained cells. The median value of all the scores was chosen as the cut-off value to divide patients into high and low expression categories [4]. All specimens were evaluated under light microscopy by two independent pathologists (M.J. and P.L.S.).

### Analysis of adenocarcinoma-associated mutations and rearrangement

Samples were analyzed for epidermal growth factor receptor (*EGFR*) mutations within exons 18 to 21 and *KRAS* (Kirsten rat sarcoma viral oncogene homolog) mutations at codons 12 and 13 using an amplification refractory mutation system (Super-ARMS EGFR Mutation Detection Kit and KRAS Mutation Detection Kit, Amoy Diagnostics Co. Ltd., Xiamen, China). The presence of anaplastic lymphoma kinase (*ALK*) and *ROS1* (ROS proto-oncogene 1, receptor tyrosine kinase) translocation was evaluated by fluorescence in situ hybridization as described previously [5, 6].

### Statistical analysis

Statistical analyses were performed using the software Statistical Package for Social Sciences, version 22.0, for Windows (SPSS, IL, USA). Chi-squared or Fisher’s exact tests were used to determine if any associations were evident between STAS and clinicopathologic parameters and the expression of immunohistochemical markers. Survival curves were determined using the Kaplan–Meier method, and statistical differences in survival times were determined using the log-rank test. The Cox proportional hazards model was applied for multivariate survival analysis. A *p* value < 0.05 was considered statistically significant.

## Results

### Patient clinicopathologic characteristics and outcome

In the cohort of 303 ADC cases, there were 150 male and 153 female patients, ranging in age from 23 to 83 years (median of 65 years). The predominant invasive pattern was acinar in 154 (50.8%), papillary in 82 (27.1%), lepidic in 45 (14.8%), solid in six (2.0%), and micropapillary in 16 (5.3%) cases. P-stage was IA in 86, IB in 87, IIA in 46, IIB in 11, IIIA in 48, IIIB in five, and IV in 20 cases. The follow-up period was from 1 to 65 months with a median of 30 months. Ninety-one patients showed recurrence, and 32 patients died of disease in the last follow-up.

In the cohort of 121 SQCC cases, patient age ranged from 31 to 85 years (median 69 years). Most patients were men (*n* = 119). P-stage was IA in 28, IB in 21, IIA in 26, IIB in 14, IIIA in 28, IIIB in one, and IV in three cases. The follow-up period was from 1 to 65 months with a median of 34 months. Thirty-two patients showed recurrence, and 16 patients died of disease in the last follow-up.

### Tumor STAS and its association with clinicopathologic parameters

**Fig. 1:**
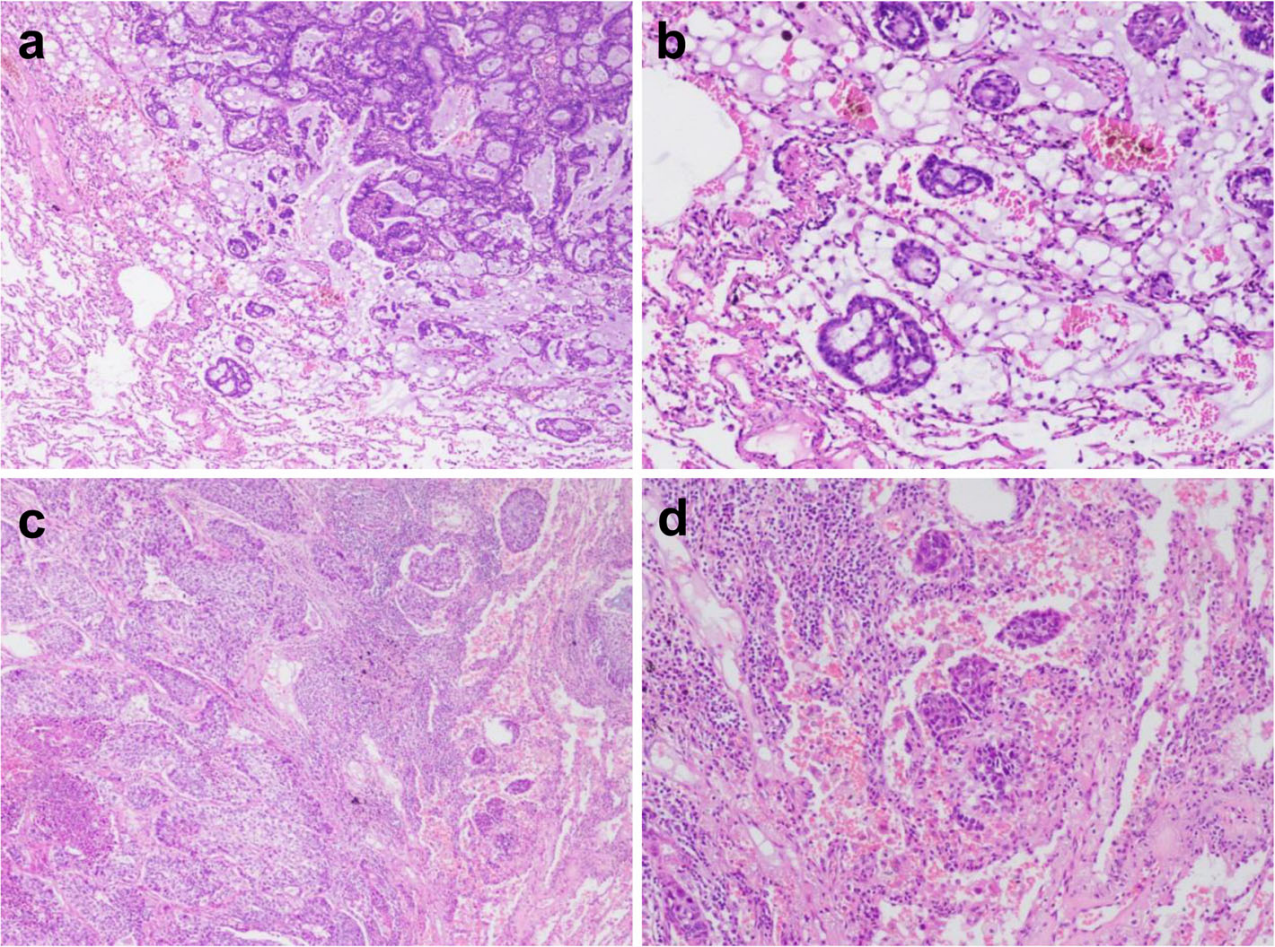
Tumor spread through air spaces (STAS). **a**, **b**: STAS in lung adenocarcinoma (ADC); **c**, **d**: STAS in squamous cell carcinoma (SQCC). (a-d: H&E staining; **a**, **c**: 40×; **b**, **d**: 100×)

In the ADC cohort, tumor STAS was observed in 183 (60.4%) cases (Fig. 1). The association between clinicopathologic parameters and STAS is summarized in Table 1. STAS was more frequently identified in tumors with pathological features characteristic of aggressive tumor behavior, such as larger tumor size (*p* = 0.002), presence of micropapillary pattern (*p* < 0.001), pleural invasion (*p* = 0.045), vascular invasion (*p* < 0.001), lymphatic invasion (*p* < 0.001), perineural invasion (*p* = 0.007), presence of tumor necrosis (*p* < 0.001), and higher p-stage (*p* = 0.003).

**Table 1:** Correlations between clinicopathological parameters and STAS in ADC

Parameters	In total	STAS		*p*
		Positive(n(%))	Negative(n(%))	
In total	303	183(60.4)	120(39.6)	
Gender				
Female	153	91(49.7)	62(51.7)	0.741
Male	150	92(50.3)	58(48.3)	
Age				
≤ 65	157	91(49.7)	66(55.0)	0.369
> 65	146	92(50.3)	54(45.0)	
Smoking history				
Non-smoker	183	112(61.2)	71(59.2)	0.723
Smoker	120	71(38.8)	49(40.8)	
Tumor size				
≤ 3 cm	177	94(51.4)	83(69.2)	0.002
> 3 cm	126	89(48.6)	37(30.8)	
Predominant subtype				
Acinar	154	89(48.6)	65(54.2)	0.104
Papillary	82	54(29.5)	28(23.3)	
Lepidic	45	27(14.8)	18(15.0)	
Solid	6	1(0.5)	5(4.2)	
Micropapillary	16	12(6.6)	4(3.3)	
Presence of micropapillary				
Absent	229	115(62.8)	114(95.0)	< 0.001
Present	74	68(37.2)	6(5.0)	
Pleural invasion				
Absent	168	93(50.8)	75(62.5)	0.045
Present	135	90(49.2)	45(37.5)	
Vascular invasion				
Absent	189	92(50.3)	97(80.8)	< 0.001
Present	114	91(49.7)	23(19.2)	
Lymphatic invasion				
Absent	148	58(31.7)	90(75.0)	< 0.001
Present	155	125(68.3)	30(25.0)	
Perineural invasion				
Absent	280	163(89.1)	117(97.5)	0.007
Present	23	20(10.9)	3(2.5)	
Tumor necrosis				
Absent	196	103(56.3)	93(77.5)	< 0.001
Present	107	80(43.7)	27(22.5)	
Tumor relapse				
Absent	212	110(60.1)	102(85.0)	< 0.001
Present	91	73(39.9)	18(15.0)	
Pathological stage				
Stage I-II	230	128(69.9)	102(85.0)	0.003*
StageIA	86	39(21.3)	47(39.2)	0.111
StageIB	87	50(27.3)	37(30.8)	
StageIIA	46	29(15.8)	17(14.2)	0.146
StageIIB	11	10(5.5)	1(0.8)	
Stage III-IV	73	55(30.1)	18(15.0)	
*EGFR* mutation				
Negative	143	96(52.5)	47(39.2)	0.023
Positive	160	87(47.5)	73(60.8)	
*KRAS* mutation				
Negative	243	148(91.9)	95(96.0)	0.201
Positive	17	13(8.1)	4(4.0)	
*ALK* rearrangement				
Negative	279	160(87.4)	119(99.2)	< 0.001
Positive	24	23(12.6)	1(0.8)	
*ROS1* rearrangement				
Negative	294	174(95.1)	120(100.0)	0.013
Positive	9	9(4.9)	0(0)	

*Correlation between stage I-II and stage III-IV

In the SQCC cohort, tumor STAS was observed in 39 (32.2%) cases (Fig. 1). The association between clinicopathologic parameters and STAS is summarized in Table 2. STAS was significantly associated with the presence of lymphatic invasion (*p* = 0.020). STAS-positive cases were more likely to show perineural invasion, although this trend was not statistically significant (*p* = 0.080). Other parameters including patient age, smoking history, tumor size, pleural invasion, vascular invasion, tumor necrosis, and p-stage showed no differences between STAS-positive and STAS-negative cases.

**Table 2:** Correlations between clinicopathological parameters and STAS in SQCC

Parameters	In total	STAS		*p*
		Positive(n(%))	Negative(n(%))	
In total	121	39(32.2)	82(67.8)	
Gender				
Female	2	0(0)	2(2.4)	1.000
Male	119	39(100.0)	80(97.6)	
Age				
≤ 65	38	15(38.5)	23(28.0)	0.249
> 65	83	24(61.5)	59(72.0)	
Smoking history				
Non-smoker	6	0(0)	6(7.3)	0.175
Smoker	115	39(100.0)	76(92.7)	
Tumor size				
≤ 3 cm	37	12(30.8)	25(30.5)	0.975
> 3 cm	84	27(69.2)	57(69.5)	
Pleural invasion				
Absent	82	28(71.8)	54(65.9)	0.513
Present	39	11(28.2)	28(34.1)	
Vascular invasion				
Absent	89	25(64.1)	64(78.0)	0.104
Present	32	14(35.9)	18(22.0)	
Lymphatic invasion				
Absent	68	16(41.0)	52(63.4)	0.020
Present	53	23(59.0)	30(36.6)	
Perineural invasion				
Absent	103	30(76.9)	73(89.0)	0.080
Present	18	9(23.1)	9(11.0)	
Tumor necrosis				
Absent	12	3(7.7)	9(11.0)	0.750
Present	109	36(92.3)	73(89.0)	
Tumor relapse				
Absent	89	29(74.4)	60(73.2)	0.890
Present	32	10(25.6)	22(26.8)	
Pathological stage				
Stage I-II	89	26(66.7)	63(76.8)	0.236*
StageIA	28	8(20.5)	20(24.4)	0.443
StageIB	21	4(10.3)	17(20.7)	
StageIIA	26	11(28.2)	15(18.3)	0.299
StageIIB	14	3(7.7)	11(13.4)	
Stage III-IV	32	13(33.3)	19(23.2)	

*Correlation between stage I-II and stage III-IV

### Tumor STAS and molecular alterations in ADC

The association between STAS and molecular alterations was exclusively analyzed in the ADC cohort (Table 1). STAS-positive cases were more likely to harbor wild-type *EGFR* (*p* = 0.023), *ALK* rearrangements (*p* < 0.001), or *ROS1* rearrangements (*p* = 0.013). *KRAS* mutations were detected in 260 cases and no correlation was found between STAS and *KRAS* mutations (*p* = 0.201).

### Tumor STAS and immunohistochemical expression

The association between STAS and immunohistochemical expression is summarized in Table 3. For both ADC and SQCC, the expression of E-cadherin and vimentin was significantly different between STAS-positive and STAS-negative cases. STAS-positive cases were more likely to show low E-cadherin expression (*p* = 0.001 and 0.012 for ADC and SQCC, respectively) and high vimentin expression (*p* = 0.003 and 0.034 for ADC and SQCC, respectively). In ADC, Ki67 expression was higher in STAS-positive cases (*p* < 0.001), whereas this correlation was not observed in SQCC. The expression of survivin was significantly higher in STAS-positive SQCC (*p* < 0.001) than in STAS-negative cases; however, this trend was not observed in ADC. The expression of Bcl-2 and Bim showed no correlation with the status of STAS in either ADC or SQCC.

**Table 3:** Correlations between immunohistochemical expression and STAS

Antibodies	In total	STAS in ADC		*p*	In total	STAS in SQCC		*p*
		Positive(n(%))	Negative(n(%))			Positive(n(%))	Negative(n(%))	
E-cadherin								
Low	171	119(66.9)	52(47.3)	0.001	32	16(41.0)	16(19.5)	0.012
High	117	59(33.1)	58(52.7)		89	23(59.0)	66(80.5)	
Vimentin								
Low	143	76(42.7)	67(60.9)	0.003	54	12(30.8)	42(51.2)	0.034
High	145	102(57.3)	43(39.1)		67	27(69.2)	40(48.8)	
Survivin								
Low	113	67(37.6)	46(41.8)	0.481	77	15(38.5)	62(75.6)	< 0.001
High	175	111(62.4)	64(58.2)		44	24(61.5)	20(24.4)	
Ki67								
Low	125	62(34.8)	63(57.3)	< 0.001	59	22(56.4)	37(45.1)	0.246
High	163	116(65.2)	47(42.7)		62	17(43.6)	45(54.9)	
Bcl-2								
Low	115	76(42.7)	39(35.5)	0.223	71	22(56.4)	49(59.8)	0.727
High	173	102(57.3)	71(64.5)		50	17(43.6)	33(40.2)	
Bim								
Low	135	87(48.9)	48(43.6)	0.387	61	23(59.0)	38(46.3)	0.194
High	153	91(51.1)	62(56.4)		60	16(41.0)	44(53.7)	

### Survival analysis

By univariate analysis, we first analyzed the association between conventional clinicopathologic factors and patient outcomes for ADC and SQCC separately. In ADC, patient age > 65, tumor size > 3 cm, the presence of pleural invasion, vascular invasion, lymphatic invasion, and more advanced p-stage were associated with a significantly worse disease-free survival (DFS) and/or overall survival (OS) (Table 4). In SQCC, the presence of lymphatic invasion and more advanced p-stage was associated with a significantly worse DFS (Additional file 2).

**Table 4:** Univariate survival analysis of DFS and OS in ADC

Parameters	DFS		OS	
	Mean DFS (month)	*p*	Mean OS (month)	*p*
Age				
≤ 65	44.34	0.228	58.92	0.033
> 65	49.46		56.52	
Tumor size				
≤ 3 cm	49.61	0.002	60.22	0.054
> 3 cm	41.87		55.70	
Pleural invasion				
Absent	51.14	< 0.001	60.15	0.002
Present	41.18		55.45	
Vascular invasion				
Absent	49.17	0.064	60.56	0.009
Present	41.21		50.79	
Lymphatic invasion				
Absent	52.00	< 0.001	59.75	0.001
Present	41.40		55.06	
Perineural invasion				
Absent	47.39	0.598	59.00	0.266
Present	43.89		52.78	
Pathological stage				
Stage I-II	49.93	< 0.001	61.47	< 0.001
Stage III-IV	37.65		47.59	
Presence of micropapillary				
Absent	48.77	0.120	58.81	0.655
Present	41.98		55.58	
STAS				
Absent	55.73	< 0.001	60.72	0.025
Present	40.42		56.79	
STAS (in Stage I-II)				
Absent	58.20	< 0.001	62.96	0.015
Present	41.91		59.57	
STAS (in Stage III-IV)				
Absent	30.75	0.736	35.30	0.332
Present	36.88		48.87	

**Fig. 2:**
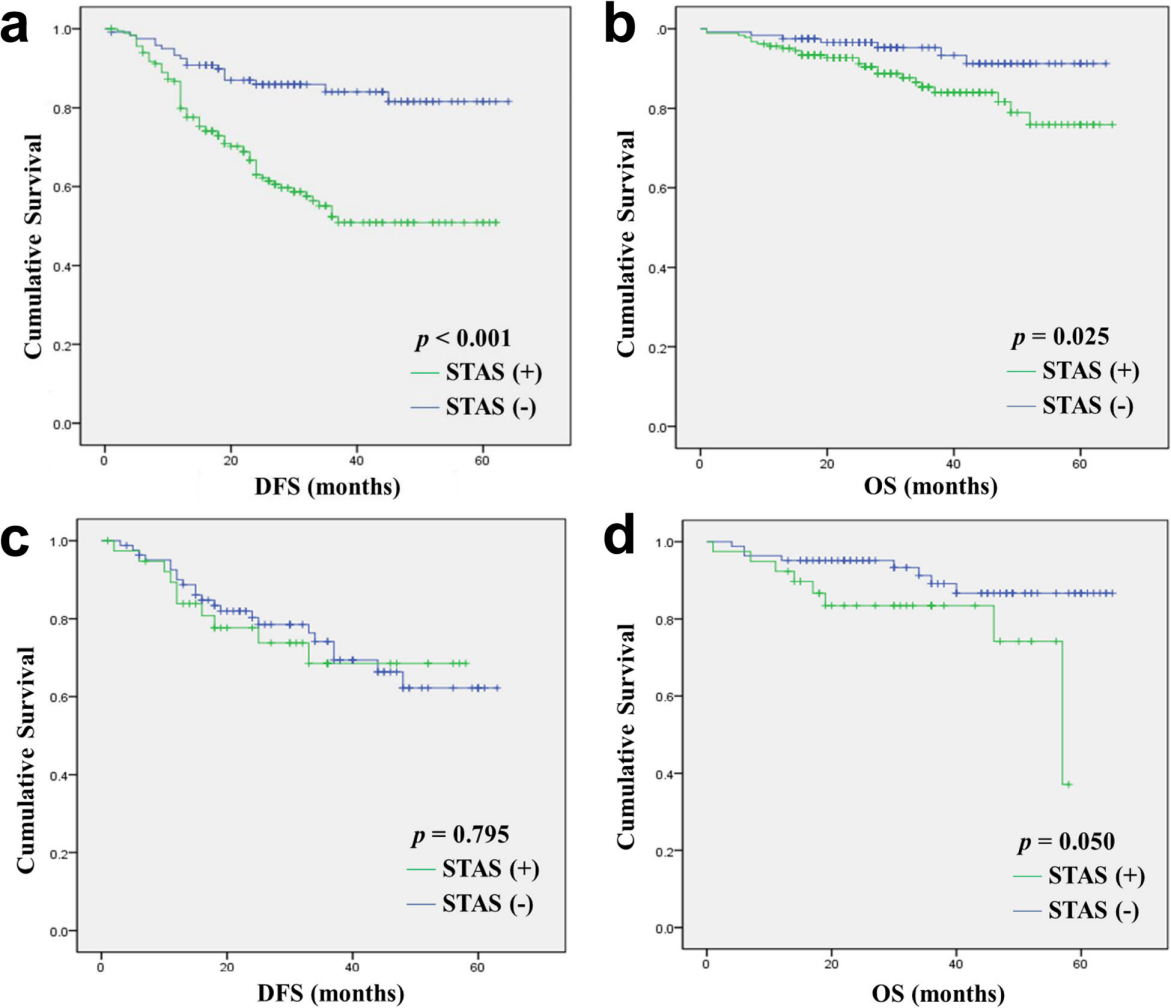
Kaplan–Meier curves according to spread through air spaces (STAS) in all-stage lung adenocarcinoma (ADC) and squamous cell carcinoma (SQCC). **a**: Disease-free survival (DFS) in ADC (*p* < 0.001); **b**: Overall survival (OS) in ADC (*p* = 0.025); **c**: DFS in SQCC (*p* = 0.795); **d**: OS in SQCC (*p* = 0.050)

Thereafter, we analyzed the prognostic significance of STAS. In ADC, STAS was associated with significantly shorter DFS (40.42 vs. 55.73 months; *p* < 0.001) and shorter OS (56.79 vs. 60.72 months; *p* = 0.025; Fig. 2, Table 4). In SQCC, STAS was associated with shorter OS, although this trend was not statistically significant (48.90 vs. 59.67 months; *p* = 0.050). STAS was not found to be associated with DFS in the SQCC cohort (44.95 vs. 48.72 months; *p* = 0.795; Fig. 2, Additional file 2). Multivariate Cox analysis showed that STAS was an independent poor prognostic factor for ADC regarding DFS but not OS (DFS: hazard ratio (HR), 2.460; 95% confidence interval (CI), 1.398–4.327; *p* = 0.002; OS: HR, 1.187; 95% CI, 0.466–3.026; *p* = 0.719; Table 5). Given the lack of a statistically significant association between clinicopathologic parameters and survival in patients with SQCC, we did not subject the outcomes of patients in this group to multivariate analyses.

**Table 5:** Multivariate Cox analysis of DFS and OS in ADC

Parameters		DFS		OS	
		*p*	HR (95% CI)	*p*	HR (95% CI)
Age	> 65 vs. ≤65	–	–	0.009	2.637 (1.275–5.455)
Tumor size	> 3 cm vs. ≤3 cm	0.137	1.383 (0.902–2.119)	–	–
Pleural invasion	Present vs. absent	0.022	1.729 (1.084–2.757)	0.158	1.878 (0.783–4.504)
Vascular invasion	Present vs. absent	–	–	0.459	1.341 (0.617–2.916)
Lymphatic invasion	Present vs. absent	0.388	1.259 (0.746–2.123)	0.289	1.792 (0.610–5.266)
STAS	Present vs. absent	0.002	2.460(1.398–4.327)	0.719	1.187 (0.466–3.026)
Pathological stage	III, IV vs. I, II	0.241	1.321 (0.830–2.102)	0.001	3.766 (1.710–8.296)

**Fig. 3:**
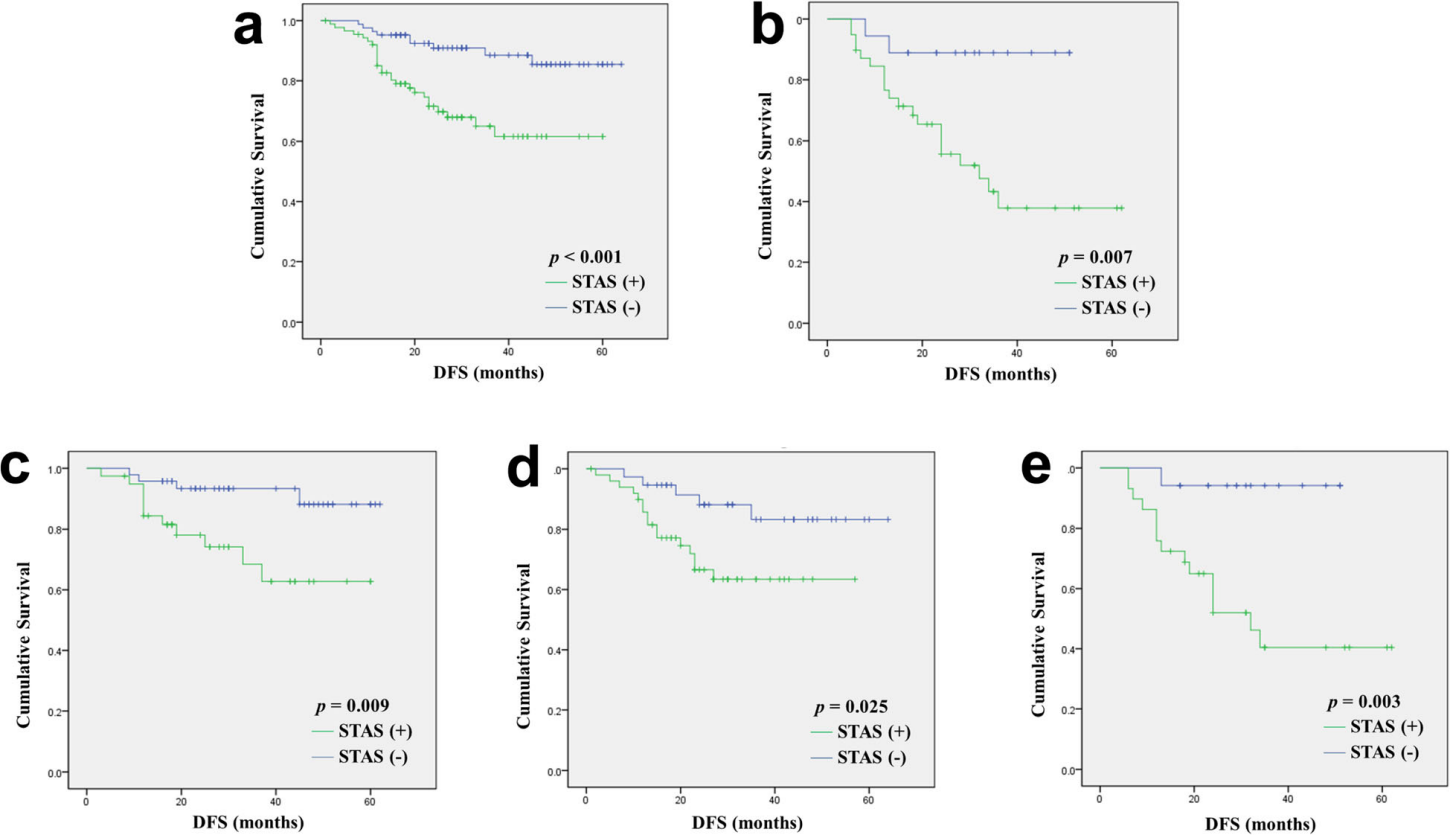
Disease-free survival (DFS) according to spread through air spaces (STAS) in single-stage lung adenocarcinoma (ADC) cases. **a**: Stage I (*p* < 0.001); **b**: stage II (*p* = 0.007); **c**: stage IA (*p* = 0.009); **d**: stage IB (*p* = 0.025); **e**: stage IIA (*p* = 0.003)

To investigate the significance of STAS in ADC of different stages, we analyzed the prognostic significance stratified by tumor stage. STAS was associated with shorter DFS and OS only in stage I-II tumors, but not in stages III-IV (DFS: *p* < 0.001 vs. *p* = 0.736; OS: *p* = 0.015 vs. *p* = 0.332; Table 4). Further stratification analysis showed that STAS was correlated with shorter DFS for stage I (*p* < 0.001), II (*p* = 0.007), IA (*p* = 0.009), IB (*p* = 0.025), and IIA (*p* = 0.003) tumors based on univariate analysis (Fig. 3, Additional file 3). However, this observation was not observed with respect to OS. In multivariate analysis, STAS was an independent risk factor for DFS in stage I cases (*p* = 0.004, Additional file 4). Multivariate analysis was not performed for stage II or IIA cases as STAS was the only risk factor for DFS. Stratification analysis was not performed for other stages of ADC or SQCC because of the small sample size in each stage.

## Discussion

In this study, we investigated the association between STAS and clinicopathologic features, molecular alterations, the expression of immunohistochemical markers, and prognostic significance in both ADC and SQCC based on Chinese patients. We found that STAS was associated with aggressive clinicopathologic features, wild-type *EGFR*, rearranged *ALK* or *ROS1*, low expression of E-cadherin and high expression of vimentin, Ki67, and survivin. In the prognostic analysis, STAS was associated with poor DFS and OS in ADC by univariate analysis and was an independent risk factor for DFS by multivariate analysis. In addition, STAS was associated with poor DFS in single stage I, II, IA, IB, and IIA ADC patients according to the new 8th edition AJCC/UICC staging system.

Since 2018, a few studies have discussed the significance of STAS based on the 8th edition AJCC/UICC staging system, and the reported results mainly focused on ADC [7–15]. Some attention has been paid to the significance of STAS in stage I patients; however, few studies analyzed the significance of STAS in other stages exclusively. Recently, Terada and colleagues found that STAS was an independent predictor of recurrence in stage III (N2) ADC [15]. In the current study, STAS was found to be associated with poor DFS and OS in stage I-II patients but not in stage III-IV cases. This observation indicates that the prognostic significance of STAS mainly exists in early-stage ADC cases, and pathological evaluation of STAS should be performed more cautiously for these tumors. In the analysis of single-stage ADC, STAS was associated with poor DFS in stage I, II, IA, IB, and IIA patients, but not OS. These results reveal more details on the significance of STAS with respect to recurrence. When STAS is observed in these lymph node-negative ADCs, close follow-up should be implemented. Further studies are needed to discuss whether these patients need post-operative adjuvant therapy.

Only a few studies have analyzed STAS in SQCC. In SQCC, the incidence of STAS was generally lower than that in ADC, which was from 19.1% [16] to 40.3% [17]. Positive STAS was observed to be associated with larger tumor size, lymphovascular invasion, tumor necrosis, high-grade tumor budding, larger nuclear diameter, higher mitotic counts, and higher T, N, and p-stages [16–18]. In survival analyses, STAS was also reported to be a significant predictive factor of DFS and OS [16–18], especially in stage I patients [16]. In the current study, STAS was associated with shorter OS, although this trend was not statistically significant, and no correlation was found between STAS and DFS. This could be because the simple size of the current study was smaller than that of previous reports.

The association between STAS and molecular characteristics has not been clearly explicated. Molecular characteristics were exclusively studied in ADC. STAS was frequently observed in tumors with *ALK* and *ROS1* rearrangements, *BRAF* mutations, or wild-type HER2 [6, 7, 19–21]. In the current study, 95.8% (23/24) cases with *ALK* rearrangements and all cases with *ROS1* rearrangements demonstrated STAS, and this observation was similar to that of previous results. Three articles reported the association between STAS and *KRAS* mutations; one study concluded that STAS was frequently observed in tumors with *KRAS* mutations, whereas the other two reported no association [7, 19, 20]. Our results also concluded no association between STAS and *KRAS* mutations. However, as the *KRAS* mutation rate is quite low in Asian patients, more data are needed to clarify this issue. Regarding *EGFR* mutations, the reported results have varied among different studies. According to Hu and colleagues, STAS is frequently observed in tumors with *EGFR* mutations [7], whereas three other studies demonstrated that STAS was associated with wild-type *EGFR* [19–21]. In contrast, in some studies, no correlation was observed between STAS and EGFR [22–24]. In the current study, STAS was observed to be associated with wild-type *EGFR*. One possible explanation for the different frequencies of STAS based on different driver gene alterations could be that STAS is more frequently observed in poorly differentiated tumors including those with a solid/micropapillary pattern [25], and *ALK* or *ROS1* rearrangements mainly exist in ADC with a predominant solid pattern [1, 26]. In contrast, STAS is also associated with a non-lepidic pattern [1, 7, 19, 20], whereas EGFR mutations were more frequently detected in ADC with lepidic growth [25].

The association between STAS and the expression of immunohistochemical markers was barely understood and the correlation between STAS and EMT has been poorly discussed. In ADC, positive STAS was reported to be significantly associated with tumor stroma metastasis-associated protein 1 expression levels [8] but was not significantly correlated with programmed death ligand 1, thyroid transcription factor 1, napsin, or CK7 expression, as well as Ki67 activity [19, 22, 23]. In the present study, STAS was found to be associated with lower E-cadherin and higher vimentin and Ki67 expression. In SQCC, previous reports concluded that STAS is associated with an increased tendency for high vimentin and Ki67 expression in comparison with levels in patients without STAS; however, the expression of p53 and E-cadherin was not associated with the status of STAS [16–18]. In the present study, STAS was found to be associated with lower E-cadherin and higher vimentin and survivin expression in the SQCC cohort. These results indicate that STAS might be more likely to be present in tumors exhibiting EMT features. EMT is a process by which epithelial cells transform into mesenchymal stem cells by losing their cell polarity and cell-to-cell adhesion and gaining migratory and invasive properties, and this process has been identified as an indicator of poor prognosis in non-small cell lung cancer [27]. Even though a relationship was found between the presence of STAS and EMT features, whether STAS cells underwent EMT remains unclear. According to Yagi and colleagues [28], the survival of STAS cells relies on blood vessel co-option, and these cells are E-cadherin-positive. This result, to some extent, challenged the opinion that STAS cells undergo EMT. In agreement with previous reports, the present results suggest that EMT might be a risk factor but not a mechanism for STAS, as tumors with EMT features were found to be more discohesive with fewer intercellular adhesions; this, it would be easier for the malignant cells to detach from the main tumor.

Our study had some limitations. On one hand, some early-stage patients in the present study received limited resection, and some patients with late-stage tumors received adjuvant therapy. These conditions might have influenced the prognosis and could affect the results of survival analysis. On the other hand, the sample size involved in the present study was small, especially for SQCC, and the patients were from one single institution.

## Conclusions

STAS is a risk factor for poor DFS and OS in lung ADC, and this significance mainly exists for early-stage (I-II) ADC cases. STAS is also associated with poor DFS for single-stage I, II, IA, IB, and IIA ADC patients. In SQCC, STAS-positive patients tended to have a poorer OS. Patients with STAS are more likely to harbor wild-type *EGFR* and rearranged *ALK* or *ROS1*. In both ADC and SQCC, STAS-positive tumors frequently showed EMT features. Our findings provide a better understanding of the implications of STAS with respect to clinicopathologic features, molecular characteristics, immunohistochemical expression, and prognosis in ADC and SQCC patients.

## Supplementary information

**Additional file 1:Supplementary Table 1.** Primary antibodies used for immunohistochemistry

**Additional file 2:Supplementary Table 2.** Univariate survival analysis of DFS and OS in SQCC.

**Additional file 3:Supplementary Table 3.** Univariate survival analysis of DFS and OS in single stage ADC.

**Additional file 4:Supplementary Table 4.** Multivariate Cox analysis of DFS in single stage ADC.

## Data Availability

The datasets used and/or analysed during the current study are available from the corresponding author on reasonable request.

## Abbreviations

ADC: Adenocarcinoma
AJCC: American Joint Committee on Cancer
ALK: anaplastic lymphoma kinase
CI: Confidence interval
DFS: Disease-free survival
EGFR: Epidermal growth factor receptor
EMT: Epithelial–mesenchymal transition
HR: Hazard ratio
KRAS: Kirsten rat sarcoma viral oncogene homolog
MIA: Minimally invasive adenocarcinoma
OS: Overall survival
p-stage: Pathological stage
ROS1: ROS proto-oncogene 1, receptor tyrosine kinase
SQCC: Squamous cell carcinoma
STAS: Spread through air spaces
UICC: Union for International Cancer Control
WHO: World Health Organization
